# Assessing outcomes of health and medical research: do we measure what counts or count what we can measure?

**DOI:** 10.1186/1743-8462-4-14

**Published:** 2007-06-28

**Authors:** Robert Wells, Judith A Whitworth

**Affiliations:** 1 Menzies Centre for Health Policy, The Australian National University, Canberra, ACT, 2601, Australia

## Abstract

Governments world wide are increasingly demanding outcome measures to evaluate research investment. Health and medical research outputs can be considered as gains in knowledge, wealth and health. Measurement of the impacts of research on health are difficult, particularly within the time frames of granting bodies. Thus evaluations often measure what can be measured, rather than what should be measured. Traditional academic metrics are insufficient to demonstrate societal benefit from public investment in health research. New approaches that consider all the benefits of research are needed.

## Background

Health and medical research in Australia received a very welcome boost in investment in the latest Commonwealth budget. The use of the word investment reflects the growing literature on the substantial economic returns to society from health and medical research (HMR). This is sometimes described as research 'payback' [1]. In 2003 Access Economics reported that return on investment for HMR in Australia was around 5-fold and that 'investment in health research and development surpasses every other source of rising living standards in our time' [2].

The Macroeconomic Commission on Health [3] concluded that the evidence is overwhelming that investments in health pay off in controlling disease, improving productivity, speeding economic growth and fostering social and political stability.

This notwithstanding, governments world wide are increasingly asking for outcome measures in the evaluation of research investment and for the justification of increased investment. Doing it well is no longer enough, and funding bodies are increasingly looking to objective ways of assessing the value of research.

In Australia, the Federal Government is planning to introduce a Research Quality Framework (RQF) to measure both the quality and impact of research undertaken within universities. As the detail of the RQF is worked through over coming months much attention will be paid to the measures of research impact [4].

With this in mind, how should the value of health and medical research best be measured?

The outputs of health and medical research are often considered under the headings of knowledge gain, wealth gain, and health gain [5-8], and it seems self evident that the latter must be the most important. That being said, the other two are very much easier to measure, so that any assessment of research worth runs the risk of falling into the trap of measuring what we can, not what is important.

### Knowledge

Knowledge gain is the least difficult to assess, albeit still problematic. This is because the outputs (usually publications) are more proximate to the research performance and can be quantified, with variable precision, by a variety of markers, in particular by bibliometrics. Thus numbers of papers, impact factors of journals, and citations are all widely used. A number of scholarly analyses have addressed the strengths, weaknesses and pitfalls of such approaches but they are relatively robust, at least within field for aggregated data. More recently new tools have been developed for bibliometric assessment of individual researchers, specifically the Hirsch index and its derivatives. Often research income is used as a surrogate, as judgments on funding usually rely on some measure of outcome. These measures, and others, as peer recognition, prizes, named lectures etc, all correlate to a degree (not surprisingly, as they all purport to measure the same thing).

But the measures given most weight by granting bodies, particularly journal impact factor, may not relate well to the ultimate goal of health and medical research, that is, health outcomes. John Cade's discovery of lithium for treatment of manic-depressive illness, unquestionably one of the greatest findings of the 20^th ^century, both in relief of suffering (health gain) and cost savings (wealth gain), was published locally [9] as were some of 2006 Nobel Laureate Barry Marshall's key early papers on Helicobacter (pyloric campylobacter) [10,11].

Journal impact factors are determined *inter alia *by citations, but in aggregate, so that they say little about individual research papers. Anecdotally some researchers claim that really original work, which overturns conventional paradigms, is not accepted by 'first rank' journals and finds its way into less prestigious publications. For example, Parish's first paper on heparanase which showed that sulfated sugars could be used to inhibit tumour metastasis by blocking heparanase action was published in 1987 in the *International Journal of Cancer *(impact factor ~ 3) [12]. The paper has been cited many times and underpinned development of the anti cancer drug PI-88 that has already been granted orphan drug status by the US Food and Drug Administration.

The use of CFSE, a fluorescent dye developed for studying cell migration and proliferation, was published in *Journal of Immunological Methods *(impact factor ~ 1.9) in 1994 [13]. The approach is used in ~20–30% of all cellular immunology papers currently published, and a *Google *search suggests that between 5,000 and 10,000 papers have been published in which the procedure is used.

*The European Journal of Hand Surgery *doubled its impact factor through an editorial review on the vagaries of citations which quoted its own papers [14].

### Wealth

Wealth generation is more difficult to measure. A range of methodologic approaches to economic assessment of health research outcome have been described both nationally and internationally. These include valuing the direct cost savings from application of research findings, the economic value of a healthy workforce from improved healthcare, economic gain from new products and technologies, and some measurement of social health gain by placing a monetary value on healthy life [8]. This review from Brunel University highlights the methologic difficulties associated with impact assessment.

Patents and formation of spin-off companies are widely used, but are only surrogates. Patents often come to nothing and many spin-offs fail. Given the significant lag time from development to commercialization for devices and diagnostics, and the very long time for therapeutic and preventive agents, wealth generation often occurs years or decades after the original research, and well outside the time frame of political decision making, or even of granting bodies.

In the health context one might ask whether a more realistic measure of wealth would identify some metric for linking savings to the health system from particular discoveries. For example, Kirschner and colleagues reported that the discovery of lithium treatment for manic-depressive disorders had saved the USA over $145 billion in hospital costs alone [15]. Extrapolating this outcome to Australia this treatment alone would result in savings in excess of Australia's national health research investment.

Another legitimate financial measure would be qualification of the contribution to economic activity from disease prevention through effective public health programs. Countries which have managed to reduce rates of tobacco consumption have, among other benefits, improved workforce productivity through reduced absenteeism.

At present there is no accepted way of including impacts of these measures in a research assessment framework.

### Health

Even more vexed, but undeniably most important, is the question of measuring health outcomes as a product of research. Given that lag times for research into practice can be very long, how is it possible to measure outcomes of investments made in time frames which match those of policy makers or granting bodies?

Measures that might be used include incorporation of research into clinical guidelines or systematic reviews of best practice, generation of such guidelines or systematic reviews, or contributions to reports (usually government reports) which inform health policy or practice. But it is hard to reduce these to metrics, and qualitative rather than quantitative approaches are needed.

Similarly, the contribution to health gain of research in health services and health systems and policy are difficult to measure. For example, the improvement in health outcomes for stroke victims treated in dedicated stroke clinics is well documented but system take-up has been poor [16]. In one sense then the impact of this work has been poor and it would therefore not rate highly on any measure of impact.

### Finding new measures of research impact

A linear approach to measuring impact that attempts to take a particular research finding and identify its health impacts will not meet most situations. In most areas, such as health services and public health, the potential for system impact will only be obvious after analysis of several studies, such as a Cochrane review, and the impetus for policy or system change might only come after governments have been persuaded of the benefits of reallocating health resources in the appropriate way. In such a scenario which piece of research legitimately claims the impact: the original study/ies? The Cochrane review? The policy 'translational' work? If all can have some claim, how is the impact 'quotient' fairly divided among them?

## Conclusion

Traditional academic metrics of research output through peer-reviewed publications and citations are insufficient to satisfy society's expectation that public investment in research results in real benefit to the society. This is particularly the case for HMR. An approach that takes into account all the benefits of research outcomes, including the freeing up of resources from savings on treatment and other costs, needs to be taken. This approach will require new metrics which are understood and accepted by society and the governments which represent it. It is no longer enough to measure what we can – we need to measure what matters.

## Competing interests

The author(s) declare that they have no competing interests.

## Authors' contributions

Both authors contributed equally to the conception of this paper, drafting the manuscript, revising it critically for important intellectual content. Both authors have read and approved the final manuscript.
